# Gold-Based Nanostructures for Antibacterial Application

**DOI:** 10.3390/ijms241210006

**Published:** 2023-06-11

**Authors:** Chinmaya Mutalik, Muhammad Saukani, Muhamad Khafid, Dyah Ika Krisnawati, Widodo, Rofik Darmayanti, Betristasia Puspitasari, Tsai-Mu Cheng, Tsung-Rong Kuo

**Affiliations:** 1 Graduate Institute of Nanomedicine and Medical Engineering, College of Biomedical Engineering, Taipei Medical University, Taipei 11031, Taiwan; d845108002@tmu.edu.tw; 2 International Ph.D. Program in Biomedical Engineering, College of Biomedical Engineering, Taipei Medical University, Taipei 11031, Taiwan; d845110002@tmu.edu.tw; 3 Department of Mechanical Engineering, Faculty of Engineering, Universitas Islam Kalimantan MAB, Banjarmasin 70124, Kalimantan Selatan, Indonesia; 4 Department of Nursing, Faculty of Nursing and Midwifery, Universitas Nahdlatul Ulama Surabaya, Surabaya 60237, East Java, Indonesia; khafid@unusa.ac.id; 5 Dharma Husada Nursing Academy, Kediri 64117, East Java, Indonesia; dyahkrisna77@gmail.com (D.I.K.); rofik.darmayanti@gmail.com (R.D.); betristasya@gmail.com (B.P.); 6 College of Information System, Universitas Nusantara PGRI, Kediri 64112, East Java, Indonesia; widodoido7@gmail.com; 7 Graduate Institute for Translational Medicine, College of Medical Science and Technology, Taipei Medical University, Taipei 11031, Taiwan; 8 Taipei Heart Institute, Taipei Medical University, Taipei 11031, Taiwan; 9 Cardiovascular Research Center, Taipei Medical University Hospital, Taipei Medical University, Taipei 11031, Taiwan; 10 Stanford Byers Center for Biodesign, Stanford University, Stanford, CA 94305, USA

**Keywords:** bacterial infection, gold nanoparticles, gold nanoclusters, gold nanorods, gold nanobipyramids, gold nanostars, antibacterial mechanism

## Abstract

Bacterial infections have become a fatal threat because of the abuse of antibiotics in the world. Various gold (Au)-based nanostructures have been extensively explored as antibacterial agents to combat bacterial infections based on their remarkable chemical and physical characteristics. Many Au-based nanostructures have been designed and their antibacterial activities and mechanisms have been further examined and demonstrated. In this review, we collected and summarized current developments of antibacterial agents of Au-based nanostructures, including Au nanoparticles (AuNPs), Au nanoclusters (AuNCs), Au nanorods (AuNRs), Au nanobipyramids (AuNBPs), and Au nanostars (AuNSs) according to their shapes, sizes, and surface modifications. The rational designs and antibacterial mechanisms of these Au-based nanostructures are further discussed. With the developments of Au-based nanostructures as novel antibacterial agents, we also provide perspectives, challenges, and opportunities for future practical clinical applications.

## 1. Introduction

Emerging nanostructures have achieved various applications in catalysis [1], biofuels [2], energy storage, and electronics [3,4,5,6,7]. Discussing specifically nanomedicines, various nanostructures have been applied for microfluidic devices [8], biosensors [9,10], drug delivery systems [11], medical imaging, disease diagnosis [12,13,14,15], and antibacterial agents [16]. With these advancements in nanomedicine, nanostructures have been intensively demonstrated to be antibacterial agents to fight contagious infections induced by bacteria [17,18]. Antibiotic abuse adds to the specific hazards posed by bacterial infections by developing antibiotic resistance, resulting in treatment failures, boosting the spread of resistant bacteria, restricting treatment alternatives, and influencing numerous medical processes. To preserve antibiotic effectiveness and limit these hazards to public health, it is critical to use antibiotics carefully and in accordance with approved prescribing guidelines [19,20,21]. Antibiotics are the main drugs to treat bacterial infections, but the overuse of antibiotics has increased the opportunity for bacterial mutations which have resulted in resistant bacteria. A systematic study estimated that the 1.27 million deaths directly resulting from bacterial antimicrobial resistance were higher than the 864,000 deaths from the human immunodeficient virus (HIV)/acquired immunodeficiency syndrome (AIDS) or 643,000 from deaths malaria in 2019 [22]. Bacterial antimicrobial resistance is a globally urgent problem that requires immediate action from health communities, including basic research and clinical medicine [23,24,25,26]. Therefore, the development of superior antibacterial nanostructures is a promising approach against bacterial antimicrobial resistance.

Semiconductor, metal, and polymer nanostructures have been extensively investigated as antibacterial agents because of their unique chemical and physical characteristics [27,28,29,30,31,32,33,34]. Among these nanostructures, metal-based nanostructures such as gold (Au), silver, platinum, and copper have been explored as antibacterial agents [35,36,37,38]. Considering their inherent toxicity, chemically functionalized silver and titanium dioxide nanoparticles have been used to demonstrate antimicrobial activity. In an alternative strategy, photo-thermal properties of metallic nanoparticles have been theoretically researched and experimentally tested against several temperature-sensitive (mesophilic) bacteria using a plasmonic-based heating therapy [39].

Recently, gold nanostructures have emerged as promising candidates in the field of biomedicine due to their unique physical and chemical properties, including excellent stability, facile modification, and high surface area-to-volume ratio [40,41,42,43,44]. Most importantly, the unique optical properties of gold nanostructures, such as surface plasmon resonance, also make these nanostructures highly attractive for various theranostic techniques, including photothermal therapy, photoacoustic imaging, and surface-enhanced Raman spectroscopy. Additionally, gold nanostructures exhibit excellent electrical conductivity and catalytic activity, enabling their use in biosensing devices and electrochemical sensors. The physical and chemical properties of gold nanostructures make them versatile tools in biomedicine.

Great achievements have proven that Au-based nanostructures with different morphologies, including Au nanoparticles (NPs), Au nanoclusters (NCs), and anisotropic Au nanostructures such as Au nanorods (NRs), Au nanobipyramids (NBPs), and Au nanostars (NSs) can serve as potential antibacterial agents owing to their outstanding structural and optical properties [45,46]. In this literature review, we collected and summarized available data on AuNPs, AuNCs, and anisotropic Au nanostructures for antibacterial applications. Furthermore, we emphasized the antibacterial mechanisms of these Au-based nanostructures to reveal their excellent antibacterial activities. Critical challenges and future perspectives of Au-based antibacterial nanostructures for fundamental investigations and clinical applications are also provided and discussed.

## 2. Antibacterial Nanostructures of AuNPs

Because of their distinctive optical and electrical characteristics, as well as their potential in numerous biomedical applications, including drug administration, imaging, and therapy, AuNPs have garnered a lot of attention in recent years [47,48,49]. The antibacterial activities of AuNPs are some of the most researched uses of these particles. We talk about current developments in the synthesis of AuNPs and their claimed mechanisms of antibacterial action in this review. Recent studies showed that AuNPs can be synthesized using various methods, including physical methods such as laser ablation and sonication, and chemical methods such as the reduction of gold ions using reducing agents. Among these methods, the most widely used method is the chemical reduction method, which involves the reduction of gold ions using reducing agents such as sodium citrate, ascorbic acid, and thiols. AuNPs can be coupled to a variety of functionalized molecules, including ligands, drugs, peptides, proteins, and so forth [50].

### Recent Advancements in the Synthesis of AuNPs and Their Antibacterial Applications

**Figure 1:**
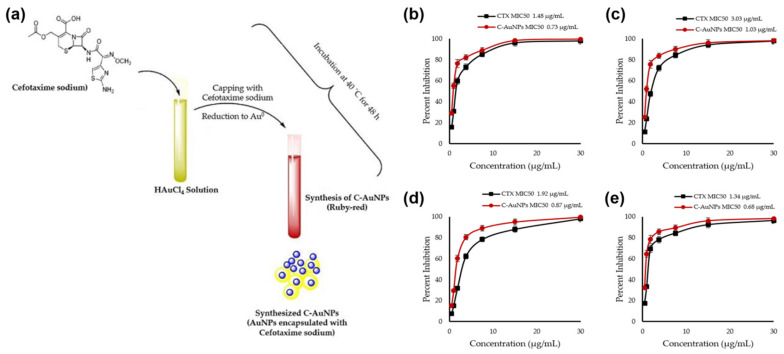
(**a**) Schematic representation of cefotaxime (CTX)-loaded gold nanoparticle (C-AuNP) synthesis and CTX and C-AuNP minimal inhibitory concentrations for (**b**) *Escherichia coli*, (**c**) *Pseudomonas aeruginosa*, (**d**) *Klebsiella oxytoca*, and (**e**) *Staphylococcus aureus*. Reproduced from ref. [51].

In recent work, cefotaxime (CTX)-loaded AuNPs (C-AuNPs) were prepared, and their antibacterial effectiveness against diverse bacterial strains was assessed. CTX is loaded onto the surface of AuNPs during their manufacture and acts as a reducing and capping agent. It was shown that C-AuNPs could be produced using a simple one-pot synthesis process (Figure 1a). By eliminating the use of external chemicals or biomolecules as reducing or capping agents, this technique prevented the development of leftover contaminants that might affect the antibacterial results. The progressive transition of the reaction solution’s hue from light yellow to ruby red after incubation with the CTX antibiotic served as evidence that C-AuNPs had successfully been synthesized. The surface plasmon resonance (SPR) that took place in C-AuNPs is responsible for this color change. Minimum inhibitory concentration (MIC_50_) values for CTX and C-AuNPs represent the concentrations that block 50% of populations of tested bacterial strains. According to Figure 1b–e, the quantified MIC_50_ values for *Staphylococcus aureus* (*S. aureus*) were 1.34 µg/mL (for CTX) and 0.68 µg/mL (for C-AuNPs), respectively. Values for *Escherichia coli* (*E. coli*), *Klebsiella oxytoca*, and *Pseudomonas aeruginosa* (*P. aeruginosa*) were 1.48, 3.03, and 1.92 µg/mL for CTX and 0.73, 1.03, and 1.87 µg/mL for C-AuNPs. In terms of the mechanism of action, C-AuNPs bind to the bacterial cell membrane and disrupt the transport of ions across the membrane, leading to a decrease in the production of adenosine triphosphate (ATP), the cell’s energy currency. The enhanced antibacterial efficacy of C-AuNPs over free CTX and AuNPs may be due to AuNPs’ substantial concentration of CTX, which is easily absorbed by bacteria and evades bacterial enzyme breakdown. Additionally, AuNPs themselves have strong antibacterial potential due to their capacity to damage bacterial DNA by direct contact and by preventing its unwinding during transcription. According to the study’s findings, a sufficient amount (83.94%) of CTX was coupled to AuNPs, which effectively delivered CTX sodium to bacterial cells. The higher concentration of CTX molecules per unit volume of the system may have contributed to the overall antibacterial activity of C-AuNPs. Increased porosity in cell walls may have allowed CTX loaded onto AuNPs to enter gram-positive bacterial cells with ease. It was also noted that AuNPs may interact with proteins and lipopolysaccharides (LPSs) on the outer membranes of gram-negative bacterial strains. This might make it easier for AuNPs to infiltrate and distribute CTX to gram-negative bacteria [51,52,53,54,55].

**Figure 2:**
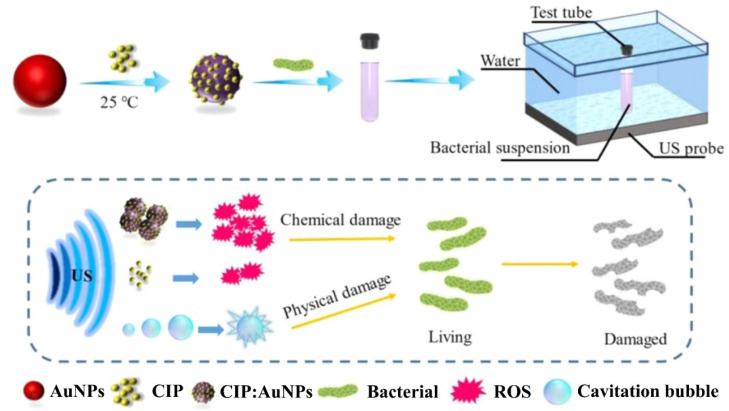
Ciprofloxacin: gold nanoparticle (CIP:AuNP) synthesis schematic and representation of the antibacterial pathway using sonodynamic antimicrobial chemotherapy (SACT). Reproduced from ref. [57].

According to a recent report, in vitro ciprofloxacin (CIP)-mediated sonodynamic antimicrobial chemotherapy (SACT) is facilitated by AuNPs. As depicted in Figure 2, 0.2 g/L AuNPs and 0.1 g/L of a CIP aqueous solution were gently stirred for 10 min of reaction time at room temperature. At 4 °C, the solution mix was centrifuged for 5 min at a speed of 12,000 rpm. The desired outcome, precipitates, could be dissolved in distilled water for subsequent experiments. Using plate colony-counting techniques, the bactericidal efficiency measurement threshold for SACT was determined. The length of ultrasonic (US) exposure, solution temperature, and CIP:AuNP concentration were all found to have significant impacts on SACT. *E. coli* and *S. aureus* were severely injured by the US, losing their usual microbial shape and revealing their contents. The aforementioned experimental findings thus originally supported the hypothesis that AuNPs could improve the bacteriostasis of CIP-mediated SACT. Additionally, it was demonstrated by intracellular reactive oxygen species (ROS) detection experiments that this acceleration may be related to ROS produced by US mechanics [56]. Research in this area usually suggests that one of the key processes explaining how US inhibits bacteria is the physical harm brought about by the cavitation effect. Scanning electron microscopy (SEM) under US irradiation allowed researchers to monitor the collapse of the membrane integrity and the efflux of cell contents from *E. coli* and *S. aureus*. The fact that CIP:AuNPs and US worked together to synergistically suppress the bacterium while the level of cell damage increased supports the idea that AuNPs might improve the cavitation impact of US by expanding nuclear sites and lowering the cavitation threshold. In addition to the physical harm caused by cavitation, the chemical impact caused by ROS also significantly contributes to the suppression of bacteria by US. When cavitation vesicles break, free radicals are created. These radicals mix with oxygen to generate ROS, which can harm cells by oxidizing proteins, lipids, DNA, and monosaccharides (Figure 2) [57,58,59,60,61,62,63].

**Figure 3:**
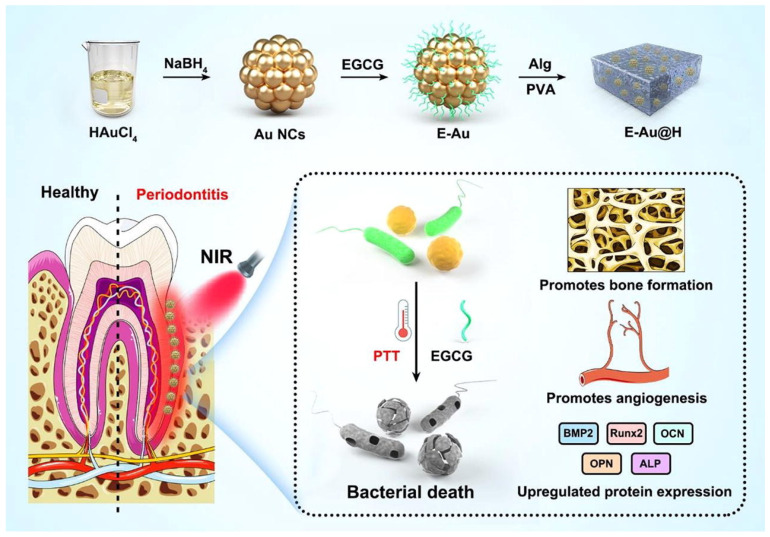
Epigallocatechin gallate (EGCG) added to hydrogels modified with gold nanoparticles (AuNPs; E-Au@H) synthesis schematic and representation of the antibacterial pathway. Reproduced from ref. [64].

Recently Dong et al. reported on adding epigallocatechin gallate (EGCG) to hydrogels modified with AuNPs (E-Au@H) to achieve combined effects of bactericidal, bactericidal photosensitization, and periodontal tissue regeneration as shown in Figure 3 [64]. In the synthesis process, a 5 mL beaker was first filled with 0.4 mL solution of HAuCl_4_ (17 mg/mL), and then the beaker was submerged in an ice-water bath. Under rapid stirring conditions (800 rpm), 2 mL of the freshly made, ice-cold NaBH_4_ solution (1.75 mg/mL) was added. Following the reaction, the product was separated by centrifugation (15 min at 15,000 rpm) and rinsed with deionized water. The cleaned product was then mixed again in 2 mL of deionized water with 1 mL of a 1 mg/mL EGCG solution, and the mixture was agitated (at 500 rpm) for 10 h at room temperature to allow for optimal adsorption. The homogenous hydrogel solution was then mixed with 0.05 g of the manufactured E-Au powder for 1 h of sonication and left to sit for 6 h before the E-Au@H product was retrieved. The mouth microbiome is interconnected with the outside environment, which can cause complicated periodontal irritation with microbial pathogens accumulating in plaque biofilms, thus making periodontal therapy very challenging. An agar plate test was used to evaluate the antibacterial efficacy of E-Au@H against gram-positive and gram-negative bacteria in order to determine whether composite materials were practical for treating periodontitis. While the near-infrared (NIR) spectrum may completely manage the release of tea polyphenols, boost the antibacterial action, promote angiogenesis, and improve osteogenesis, E-Au@H swiftly heated up to 50.7 °C in less than 5 min when exposed to NIR light. The NIR-irradiated nanocomposite inhibited *S. aureus*, *E. coli*, and *S. aureus* biofilms by 94%, 92%, and 74%, respectively, according to in vitro studies. They also increased alkaline phosphatase activity 5-fold after 7 days and the rate of extracellular matrix mineralization by 3-fold after 21 days. E-Au@H with NIR laser illumination was shown to reduce dental plaque biofilms by 87% in an animal model [64,65,66,67,68].

**Figure 4:**
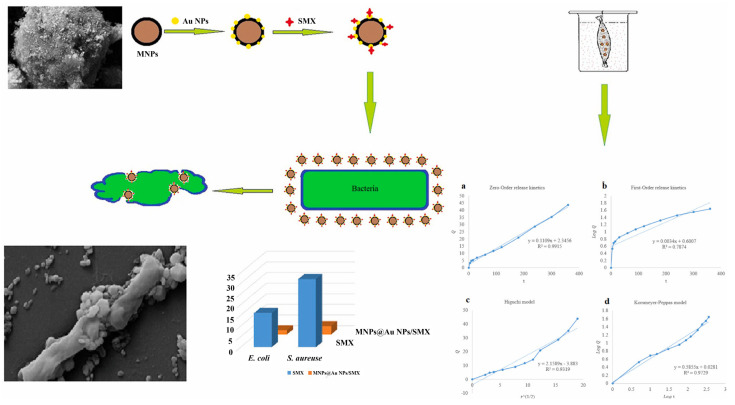
Sulfomethoxazole (SMX)-conjugated magnetic and gold nanoparticle (AuNP) synthesis and antibacterial studies. Reproduced with permission from ref. [69]. Copyright 2022 Elsevier.

A recent paper described a straightforward procedure for creating customized gold-decorated magnetic NPs with a high capacity to alter conventional formulations of antibiotics like sulfamethoxazole (SMX) and a subsequent investigation of the drug’s adsorption-desorption (release) cycle. Several techniques, including zeta potential testing and field emission scanning electron microscopy, were used to analyze these produced NPs. AuNPs decorated with magnetic NPs on SMX bind to the cell membranes of bacteria and impair ion transport across the membrane, resulting in a decrease in ATP synthesis, the cell’s energy currency. This results in the destruction of bacterial cell walls, and there is a possibility of ROS generation, leading to microbial elimination. In this study, there was a unique representation of an antibacterial activity and mechanism that was associated with the study of material kinetics and dynamics. Temkin and pseudo-first-order Lagergren models were respectively utilized to study the drug sorbate’s adsorption isotherms and kinetics; a zero-order model was used to study the kinetics of the drug’s release from this carrier. MIC values of pure SMX and SMX-conjugated magnetic NPs and AuNPs in antibacterial tests were calculated to be 14 and 2.5 µg/mL against *E. coli* and 24 and 1.25 µg/mL against *S. aureus*, respectively, displaying the highest order of antibacterial activity (Figure 4) [69,70,71,72,73,74].

**Figure 5:**
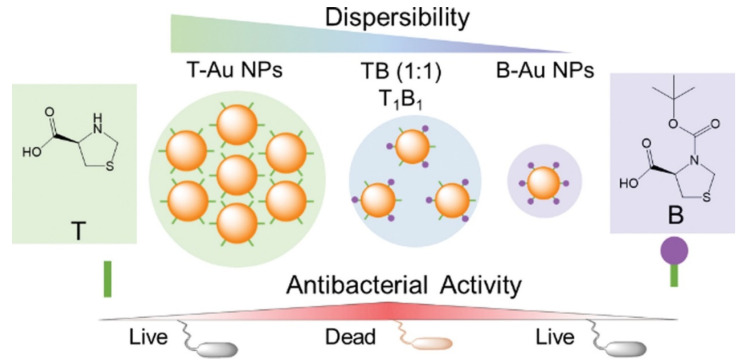
The synthesis approach of thioproline (T) (Boc-protected (B))-gold nanoparticles (AuNPs) to modify their antibacterial activity as schematically depicted. Reproduced with permission from ref. [75]. Copyright 2022 Royal Society of Chemistry.

Another mechanism of AuNPs is affected by the particle dispersibility, size, and shape, as well as the presence of other molecules such as oxygen, water, and organic substances. Following the above-mentioned trial, one recent study reported that aggregation was a major element impacting NPs’ functions using thioproline (T) and Boc-protected thioproline (B) in combination with AuNPs [74]. The antibacterial activity of thioproline-modified AuNPs attained its minimum potential by thioproline-mediated particle aggregation. The maximum antimicrobial activities of AuNPs were attained by fine-tuning the balance between thioproline exposure and shielding. A strategy for balancing NP monodispersity with antibacterial activity was described. Maximum antibacterial properties were obtained by fine-tuning the equilibrium between the exposition and sheltering of active chemicals on AuNPs (Figure 5). The AuNPs synthesized in this study were altered with organic substance concentrations to describe the mechanistic antibacterial behavior of functionally modified AuNPs. The study was conducted using multidrug-resistant (MDR) and non-MDR *E. coli* and *Klebsiella pneumonia*, and many more gram-negative bacteria were found to be affected by T_1_B_1_ (1:1)-AuNPs, which showed the highest antibacterial activity in a subsequent study. T_1_B_1_-AuNPs promoted cell membrane permeability and, hence, killed bacteria. Although the negative charge of T_1_B_1_-AuNPs may prevent them from interacting with negatively charged bacteria, the carboxylic acid groups of T ligands might compete for hydrogen bonding interactions with LPSs/peptidoglycans on bacterial surfaces. This action may disrupt hydrogen bonds within the cell wall, causing instability and disintegration of the cell walls, which is a plausible mechanism for T_1_B_1_-AuNPs’ antibacterial activity [75,76,77,78,79].

## 3. Antibacterial Nanostructures of AuNCs

**Figure 6:**
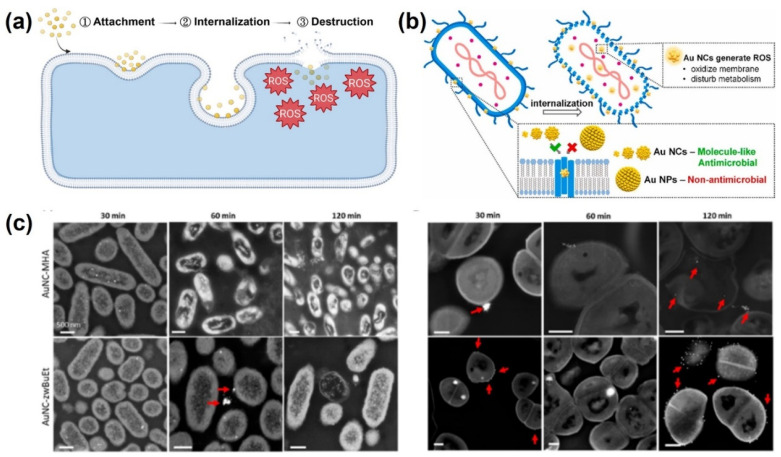
(**a**) Stages of antibacterial mechanism of gold nanoclusters (AuNCs). Reproduced with permission from ref. [84]. Copyright 2022 American Chemical Society. (**b**) Illustration of bacterial eradication by AuNCs. Reproduced with permission from ref. [88]. Copyright Elsevier 2021. (**c**) Interaction analysis of bacteria and AuNCs at different time points. Reproduced with permission from ref. [91]. Copyright 2022 American Chemical Society.

AuNCs consist of several to hundreds of gold atoms with a size of <3 nm which are protected by organic ligands [80]. AuNCs, or ultrasmall AuNPs, are promising nanostructures that have unique molecule-like properties for several applications, including theranostic, antibacterial, sensing, and catalysis [81,82,83]. Au-based NPs are known as materials with good biocompatibility with mammalian cells even if the size is reduced to NC size [84,85,86]. AuNPs with particle sizes of >3 nm are inert for bacteria, whereas AuNCs have shown potent antibacterial activity [87]. AuNCs have shown effective antibacterial activity due to their positive charge, while the bacteria have a negative charge, which can help the NCs penetrate cell membranes. In addition, the ultrasmall size of AuNCs makes it easier for them to internalize into bacteria, thereby facilitating the process of bacterial inhibition. The antibacterial mechanism of AuNCs against bacteria is complex and not fully understood. Generally, the antibacterial mechanism of AuNCs includes attachment, internalization, and destruction of bacterial membranes through ROS reactions [84]. Ultrasmall AuNCs are able to readily pass through cell wall pores and become internalized in bacteria, where they induce the production of ROS, which oxidize bacterial membranes and disrupt bacterial metabolic processes [88]. The antibacterial mechanism was investigated at the cellular level through the destruction of membrane integrity, disruption of the antioxidant defense system, metabolic disturbances, and DNA damage (Figure 6), and also at the molecular level through a transcriptome analysis (RNA sequencing) [89]. Furthermore, AuNCs have exhibited antibacterial mechanisms of photodynamic therapy (PDT) and photothermal therapy (PTT). Therefore, AuNCs have strong antibacterial activity against a wide range of bacterial strains, including both gram-positive and gram-negative bacteria, MDR bacteria, and even bacterial biofilms [90,91,92,93,94].

### 3.1. Antibacterial AuNCs for Gram-Positive and Gram-Negative Bacteria

**Figure 7:**
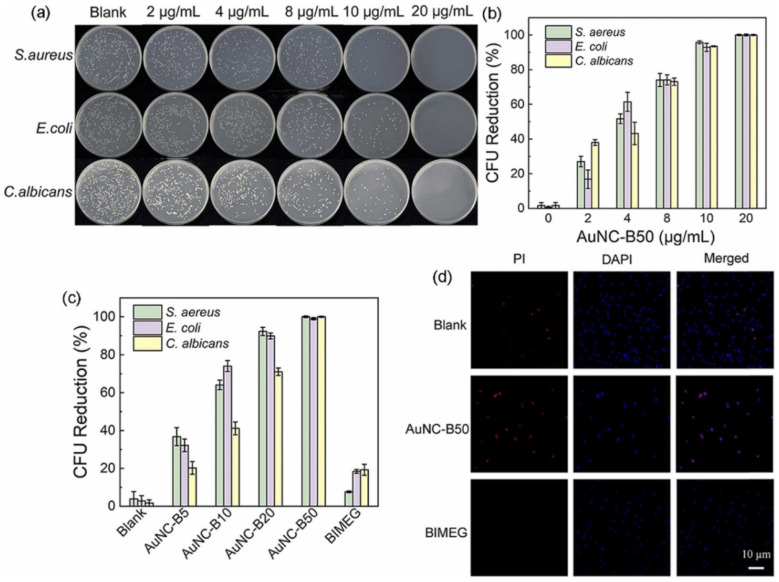
Antibacterial performance of gold nanoclusters (AuNCs) with the N-heterocyclic carbene ligand. (**a**) Photographs of agar plates and (**b**) colony forming unit (CFU) reduction rates of *S. aureus*, *E. coli*, and *C. albicans* incubated with AuNCB50 at various concentrations in the dark for 10 min. (**c**) CFU reduction rates of *S. aureus*, *E. coli*, and *C. albicans* incubated with AuNCs at different ligand ratios. (**d**) Live/dead staining of *S. aureus* before and after incubation with AuNC-B50 (20 µg/mL) for 30 min. Reproduced with permission from ref. [86]. Copyright 2022 Elsevier.

Gram-positive and gram-negative bacteria are two major categories of bacteria based on their cell wall structure. Gram-positive bacteria have a thick peptidoglycan cell wall, whereas gram-negative bacteria have a thinner peptidoglycan layer and an outer membrane containing LPSs. Gram-negative bacteria are generally considered more resistant to antibiotics and other antimicrobial agents than are gram-positive bacteria [93]. Both kinds of bacteria can effectively be killed by AuNCs. Ligands are an important factor that can improve the antibacterial capability of AuNCs. Au_25_MHA_18_ (Au_25_ protected by the 6-mercaptohexanoic acid (MHA) ligand) effectively killed ~95% of *S. aureus* and ~96% of *E. coli* after 2 h of treatment with increases in ROS levels that reached 2~3-fold for *S. epidermidis*, *Bacillus subtilis*, *E. coli*, and *P. aeruginosa* [87,88,91]. AuNCs conjugated with cysteine (Cys-AuNCs) also increased ROS levels 2.1-fold in *E. coli,* whereas (11-mercaptoundecyl)-N,N,N-trimethylammonium bromide (MUTAB)-conjugated AuNCs increased ROS levels 5-fold [88]. In addition to effectively killing gram-positive and gram-negative bacteria, AuNCs eradicated fungi (*Candida albicans*) [94] with the N-heterocyclic carbene (NHC) ligand, with a reduction in the colony-forming unit (CFU) rate of more than 99% at a concentration of 20 µg/mL (Figure 7) [86].

### 3.2. Antibacterial AuNCs for MDR Bacteria

**Figure 8:**
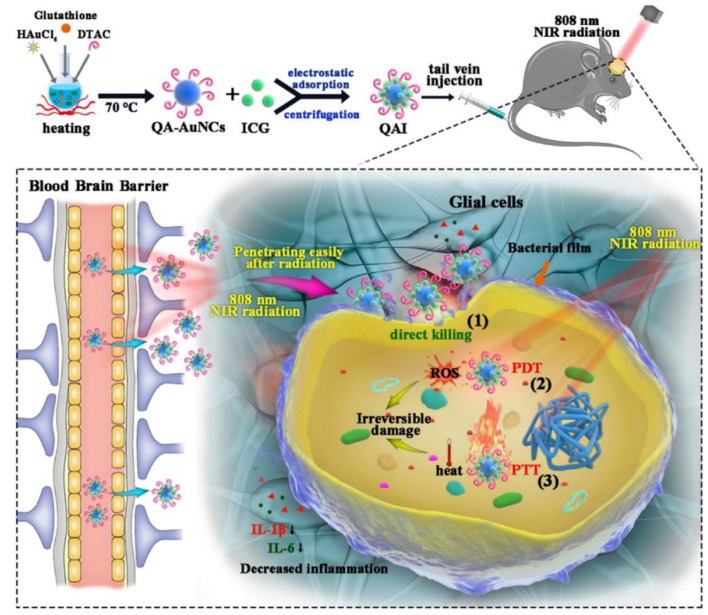
Quaternary ammonium (QA)-gold nanoclusters (AuNCs) conjugated with indocyanine green (ICG) revealed triple weapons, including direct killing, photodynamic therapy (PDT), and photothermal therapy (PTT) for methicillin-resistant *Staphylococcus aureus* (MRSA) eradication under near-infrared (NIR) irradiation. Reproduced with permission from ref. [92]. Copyright 2022 Elsevier.

MDR bacteria are strains of bacteria that are resistant to multiple types of antibiotics. These bacteria have developed mechanisms to protect themselves from the effects of antibiotics, making it difficult to treat the infections they cause. One of the ways that AuNCs can fight MDR bacteria is through the disruption of bacterial cell membranes. The small size and high surface-area-to-volume ratio of AuNCs allow them to penetrate cell membranes and disrupt their integrity, leading to bacterial cell death. AuNCs functionalized with quaternary ammonium (QA) salt (QA-AuNCs) were designed to combat methicillin-resistant *S. aureus* (MRSA). In vitro and in vivo studies showed their excellent performance as a safe and efficient strategy for clinical treatment of MRSA [89]. In addition, the gram-positive bacterium, *Clostridium difficile*, was inhibited by MHA-AuNCs. MHA-AuNCs (100 µM) generated a 5-fold increase in ROS levels, which drastically killed *C. difficile* by destroying membrane integrity without toxic effects on human cells [90]. Not only MDR gram-positive strains can be eradicated when interacting with AuNCs, but also MDR gram-negative strains. MUTAB-AuNCs were reported to eradicate vancomycin-resistant enterococci from MDR isolates [94]. AuNC-Mal/TU effectively inhibited MDR *P. aeruginosa* after 15 min of incubation. The antibacterial mechanisms of AuNC-Mal/TU were proven through multiple modes of actions in bacteria, such as inhibiting the TrxR enzyme, depleting ATP, and interfering with copper regulation [90]. Enhanced ROS generation by AuNCs and the overcoming of poor drug penetration can be induced by light irradiation. Under NIR 405-nm light irradiation, AuNCs combined with lysozyme and curcumin (Lys-AuNCs-Cur) destroyed the integrity of the outer membranes of MRSA bacteria with a killing rate of 99.92% [91]. Zhuo et al. demonstrated QA-AuNCs with a positive surface charge and an average size of ~2 nm which efficiently bound to bacteria. With NIR irradiation, QA-AuNCs can be easily internalized by bacteria and then accelerate ROS generation and toxic hyperthermia to result in bacterial eradication with triple mechanisms, including direct killing, PDT, and PTT (Figure 8). With irradiation for 10 min, QA-AuNCs at a low concentration (50 µg/mL) eradicated MRSA in biofilms without developing resistance, and decreased inflammation [92].

### 3.3. Bacterial Biofilms

**Figure 9:**
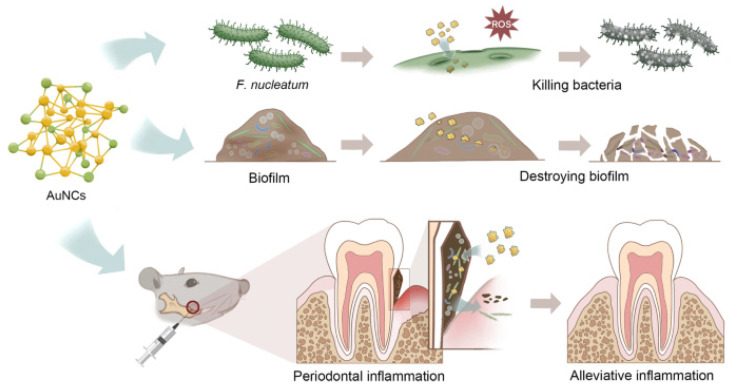
Excellent ability of gold nanoclusters (AuNCs) to fix problems due to oral biofilms. Reproduced from ref. [96].

In the medical field, bacterial biofilms can form on a variety of surfaces, including medical devices such as catheters, artificial joints, and dental implants. These biofilms can cause infections that are difficult to treat because the bacteria are protected by extracellular polymeric substances, making them less susceptible to antibiotics and the host’s immune response. The use of conventional antibiotics to treat bacterial biofilms can lead to the development of MDR. Therefore, ultrasmall AuNCs are a promising material which can combat bacterial biofilms. AuNCs were found to be able to penetrate biofilms and disrupt the structural integrity of the matrix, allowing antibiotics to reach the bacteria more effectively. *Enterococcus faecium*, *K. pneumoniae*, *Acinetobacter baumannii*, *S. aureus*, *P. aeruginosa*, and *Enterobacter* species are often found in biofilms and are major microorganisms that adhere to surfaces and form a protective matrix around themselves. AuNCs protected by mercaptopropionic acid (MPA-AuNCs) exhibited electronegative properties and revealed a 10-fold higher cellular internalization than AuNCs protected with glutathione (GSH-AuNCs) [79]. Surface chemical modifications were conducted by Srinivasulu et al. to utilize MPA-AuNCs as an antibiofilm nanostructure [95]. MPA-AuNCs further conjugated with protoporphyrin IX (PpIX) and chitosan to form PpIX-Chito-Au18 nanocomposites were used to destroy biofilms of *S. aureus* and *P. aeruginosa*. Under white light irradiation, the PpIX-Chito-Au18 nanocomposites demonstrated better biofilm penetration and elimination capacity of biofilms, including *S. aureus* and *P. aeruginosa*, compared to that of nanocomposites without white light irradiation. *Fusobacterium nucleatum* is known to be a keystone species in the formation of oral biofilms, particularly in periodontal diseases such as gingivitis and periodontitis. Ultrasmall MHA-AuNCs showed superior antibacterial effects against oral biofilms of *F. nucleatum* due to their effective penetration and destruction. In vivo studies confirmed that periodontal inflammation and bone loss may be reduced after topical application of MHA-AuNCs, because MHA-AuNCs destroyed the biofilms caused by the *F. nucleatum* bacterium. Microbiome investigations also showed that MHA-AuNCs could fix the damage in the mouth and gut microbiota caused by infection with *F. nucleatum* (Figure 9) [96].

## 4. Anisotropic Au-Based Nanostructures as Antibacterial Agents

Anisotropic Au-based nanostructures have size- and shape-dependent chemical and physical features [97,98,99]. Currently, various Au-based nanostructures have been designed and prepared, including NRs, NBPs, NSs, nanowires, triangles, cubes, octahedrons, and plates. Studies have reported uses of various Au-based nanostructures with exceptional antibacterial activities. Herein, we focused on Au-based nanostructures for highly frequent utilization as antibacterial agents, including AuNRs, AuNBPs, and AuNSs. The antibacterial mechanisms of antibacterial nanostructures of AuNRs, AuNBPs, and AuNSs are also emphasized to disclose their future perspectives.

### 4.1. Nanostructures of AuNRs for Antibacterial Applications

**Figure 10:**
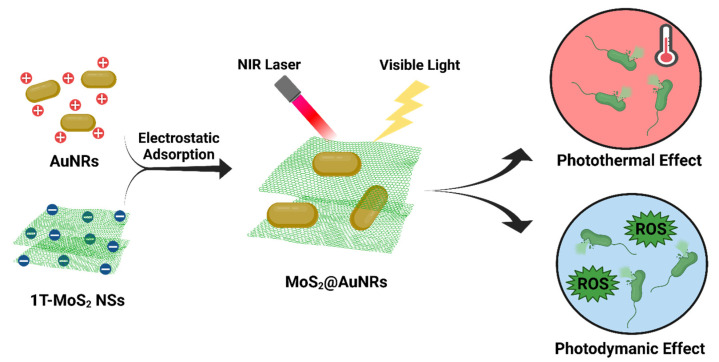
Illustration of the preparation of molybdenum-disulfide-conjugated gold nanorod (MoS_2_@AuNR) nanocomposites and their antibacterial application based on photothermal therapy (PTT) and photodynamic therapy (PDT). Reproduced from ref. [103].

Anisotropic AuNRs have achieved several antibacterial applications because of their unique surface plasmon resonance (SPR) [100]. AuNRs have two SPR absorptions, including transverse and longitudinal surface plasma absorptions. Based on their SPR, AuNRs can interact with incident light and transform light energy into heat for PTT. A recent study reported the antibacterial activities of AuNRs636 (with a longitudinal plasmon peak at 636 nm), AuNRs772 (with a longitudinal plasmon peak at 772 m), AuNPs, and AgNPs under incandescent light illumination [101]. With white light illumination, AuNR636 and AuNRs772 exhibited significant antibacterial activities, whereas AuNPs had no significant antibacterial activity against *E. coli*, *S. aureus*, *Salmonella enterica* serovar typhimurium, or MRSA. Furthermore, compared to AuNRs772, AuNRs636 presented higher antibacterial activity due to higher dangling bonds. Most importantly, under white light illumination, both AuNRs772 and AuNRs636 were proven to express PTT and PDT because of their damage to bacterial cell membranes, which decreases the cell membrane potential of bacteria and increases DNA degradation. Plasmonic photothermal therapy (PPTT) is a non-invasive and drug-free treatment method that utilizes the unique properties of noble metal nanoparticles to convert bio-transparent electromagnetic radiation into heat. When subjected to resonant laser irradiation, gold nanorods (GNRs) become highly efficient nano-converters, effectively generating heat for PPTT applications. In this study, the goal was to evaluate the antimicrobial impact of easily synthesizable, purified, and water-dispersible GNRs on *E. coli* bacteria. It was crucial to control the concentration of GNRs used in the process to avoid cytotoxic effects on cells while still producing sufficient heat, under near-infrared illumination, to raise the temperature to approximately 50 °C within approximately 5 min. Viability experiments demonstrated that the proposed system achieved a killing efficiency capable of reducing the population of Escherichia coli by approximately 2 log CFU (colony-forming unit) [102]. In our previous work, visible-light-activated metallic molybdenum disulfide nanosheets (1T-MoS_2_ NSs) and AuNRs with plasmonic absorption at a wavelength of 808 nm were combined to form nanocomposites by electrostatic adsorption. 1T-MoS_2_ NSs were synthesized through a solvothermal method. Aqueous solutions of AuNRs at three different concentrations, namely 100 µg/mL, 50 µg/mL, and 33.3 µg/mL, were prepared in CTAB aqueous solutions (125 µM). For the preparation of AuNR-decorated 1T-MoS_2_ NSs, three samples of the 1T-MoS_2_ NS solution (500 µL, 100 µg/mL) were combined with 500 µL of AuNR solutions with varying concentrations: 100 µg/mL (MoS_2_@AuNRs), 50 µg/mL (MoS_2_@1/2AuNRs), and 33.3 µg/mL (MoS2@1/3AuNRs). The photothermal properties were examined using an 808 nm NIR laser for MoS_2_@1/2AuNRs. The total amount of reactive oxygen species (ROS) generated was determined by measuring the fluorescence intensity produced by mixing 1 mL of the working solution with 200 µL of each sample. The antimicrobial phototherapy effect of MoS_2_@AuNRs on *E. coli* was assessed using the agar plate counting method. After exposing the bacterial samples to NIR laser irradiation for 2 min, the MoS_2_@1/3AuNRs, MoS_2_@1/2AuNRs, and MoS_2_@AuNRs achieved bacterial reductions of 84.4%, 97.5%, and 99.0%, respectively. Additionally, when subjected to visible light irradiation for 1 min, the antibacterial rates of MoS_2_@1/3AuNRs, MoS_2_@1/2AuNRs, and MoS_2_@AuNRs were found to be 83.8%, 93.3%, and 98.5%, respectively [103]. Under 808-nm NIR laser irradiation for 10 min, the temperature of the MoS_2_@AuNR nanocomposites increased from 25 to 66.7 ºC based on a photothermal effect. Moreover, MoS_2_@AuNR nanocomposites revealed a capability to generate ROS under visible light irradiation due to a photodynamic effect. With the combination of PTT and PDT, the MoS_2_@AuNR nanocomposites exhibited higher antibacterial activity compared to that of only PTT or PDT (Figure 10). To sum up, light-activated MoS_2_@AuNR nanocomposites showed a brilliant synergistic effect of PTT and PDT to provide an alternative approach to fight bacterial infections.

### 4.2. Nanostructures of AuNBPs for Antibacterial Applications

**Figure 11:**
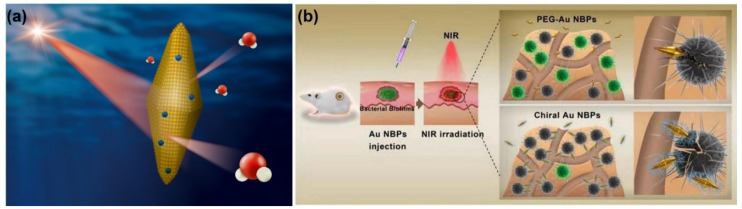
(**a**) Schematic illustration of gold nanobipyramids (AuNBPs) with the (111) plane for water desorption under near-infrared (NIR) laser irradiation. Reproduced with permission from ref. [105]. Copyright 2021 Elsevier. (**b**) Evaluation of in vivo antibacterial activity and antibacterial mechanisms of AuNBPs functionalized with chiral glutamic acid (D/L-Glu-AuNBPs). Reproduced with permission from ref. [106]. Copyright 2020 Elsevier.

Extensive explorations of plasmonic AuNBPs for the photothermal killing of bacteria have significantly impacted the development of antibacterial agents in recent years [104]. In our recent work, the photothermal effects of AuNBPs and AuNRs with similar longitudinal surface plasma bands at ~808 nm were compared under NIR laser irradiation [105]. With 808-nm laser irradiation, AuNBPs with the (111) plane exhibited a better photothermal effect than that of AuNRs with the (200) plane. According to density function theory simulations, the water adsorption energy of Au(111) is higher than that of Au(100). Therefore, based on simulations, water molecules can more easily desorb from AuNBP surfaces for photothermal heating compared to AuNRs (Figure 11a). Furthermore, for PTT, AuNBPs exhibited higher antibacterial activity than did AuNRs, according to the growth rates of *E. coli*. In combinations of experimental PTT and DFT simulations, AuNBPs were found to be a potential antibacterial agent for noninvasive PTT. Moreover, AuNBPs were functionalized with chiral glutamic acid (D/L-Glu-AuNBPs) for PTT against bacteria and biofilms [106]. Based on chemical and physical interactions with bacteria, D/L-Glu-AuNBPs were enhanced to target and interact with bacterial cell walls. With sharp tips and a nanoscale size, AuNBPs can easily penetrate bacteria and biofilms and further can induce damage to bacterial cell walls and cause leakage of bacterial components. D/L-Glu-AuNBPs also showed enhancement of DNA and nucleic acid leakage under NIR laser irradiation. In vitro and in vivo antibacterial and antibiofilm investigations demonstrated remarkable performances of D/L-Glu-AuNBPs in killing bacteria and eradicating biofilms based on synergistic effects, including chemotherapy, physiotherapy, and PTT. Overall, AuNBPs and their nanocomposites provide a novel strategy to fight bacteria and biofilms.

### 4.3. Nanostructures of AuNSs for Antibacterial Applications

**Figure 12:**
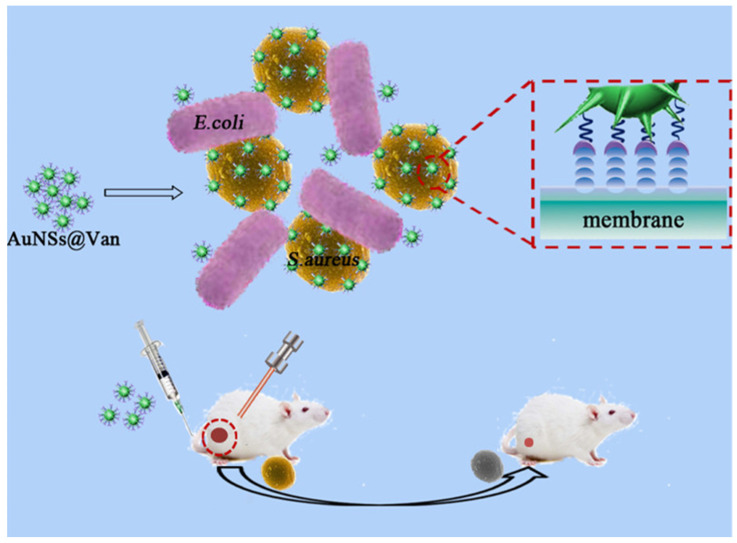
Schematic Illustration of vancomycin-coated gold nanostars (AuNSs@Van) for targeting and killing methicillin-resistant *Staphylococcus aureus* (MRSA) under near-infrared (NIR) laser irradiation. Reproduced with permission from ref. [109]. Copyright 2019 American Chemical Society.

Au-based nanostructures with special star structures, AuNSs, were proven to have excellent antibacterial effects. AuNSs have a spiked structure that can help disrupt outer bacterial cell membranes, leading to the death of bacteria. A recent study showed that AuNPs (250 μg/mL) cultured with *S. aureus* revealed no antibacterial effect [107]. However, at the same concentration (250 μg/mL), AuNSs exhibited an obvious antibacterial effect against *S. aureus*. The antibacterial mechanism of AuNSs can be attributed to the high specific surface area (surface area/volume) and star-shaped spikes of AuNSs, which induce high local stress for bacterial cell membranes, resulting in the rupture of bacterial cell membranes. Most importantly, AuNSs with unique SPR can provide superior NIR photothermal conversion performance [108]. A recent achievement was to prepare vancomycin-coated AuNSs (AuNSs@Van) for selective targeting of MRSA and elimination of MRSA under NIR laser illumination (Figure 12) [109]. For in vivo studies, AuNSs@Van showed excellent biocompatibility and outstanding antibacterial activity against bacterial infections based on physical effects in a mouse model. Furthermore, AuNSs@Van were proven to be a potential antibacterial agent for PTT in fighting gram-positive bacteria. For future health care, AuNS-based antibacterial agents have revealed promising abilities as nanoantibiotics. Moreover, in Table 1, we have summarized gold-based nanostructures and their antibacterial mechanisms in this review.

## 5. Conclusions

In this review, recent achievements of Au-based nanostructures for antibacterial applications were collected and summarized according to their size and shape. Au-based nanostructures, including AuNPs, AuNCs, and AuNBPs, have been intensively utilized as antibacterial agents according to their antibacterial mechanisms, such as increasing ROS generation, PTT, PDT, and physical interactions. These Au-based nanostructures have revealed great potential for antibacterial applications in food conservation, wastewater depuration, and bacterial infections in humans. Combined with two or more antibacterial mechanisms, Au-based nanostructures have shown brilliant bactericidal effects. Although various Au-based nanostructures have been demonstrated as antibacterial agents, their performances of bacterial inactivation still need to be increased. For clinical treatment, antibacterial nanostructures have to reach 99.99% inhibition of bacterial growth. For future perspectives, to increase antibacterial activity, the rational design of Au-based nanostructures could focus on combining various antibacterial mechanisms based on their synergistic effects. Furthermore, with surface modifications, Au-based nanostructures can be conjugated with bactericidal motifs such as ligands and antibiotics. Eventually, for real clinical applications, the biocompatibility and elimination of Au-based antibacterial nanostructures in the human body should be investigated and improved. Gold nanostructures have gained significant attention in recent years due to their unique properties and potential applications in various fields, including medicine. As antibacterial agents, gold nanostructures offer several advantages, such as their small size, high surface-area-to-volume ratio, and ability to be easily functionalized with antibacterial agents. Although gold is generally considered biocompatible, the toxicity of gold nanostructures can vary depending on their size, shape, surface charge, and surface functionalization. It is essential to carefully evaluate the potential toxic effects of these nanostructures on both bacteria and human cells to ensure their safety and minimize any harmful side effects. Other challenges include understanding the precise mechanisms by which gold nanostructures exert their antibacterial activity, which is crucial. While it is believed that their small size and large surface area contribute to enhanced antimicrobial properties, the specific interactions between gold nanostructures and bacteria, such as membrane disruption or oxidative stress induction, need to be elucidated to optimize their antibacterial efficacy. On the positive side is their use to treat bacterial biofilms, which pose a significant challenge in healthcare settings, as they exhibit increased resistance to conventional antibiotics. Gold nanostructures have shown potential in disrupting biofilms and preventing their formation. Further research is needed to develop effective strategies for eradicating biofilms using gold nanostructures. Gold nanostructures have unique optical properties that can be harnessed for diagnostic purposes, such as biosensing or imaging. Additionally, they can serve as drug delivery vehicles, facilitating the targeted and controlled release of therapeutic agents at the site of infection. Human clinical studies of Au-based antibacterial nanostructures are a critical procedure for future practical applications in medicine. With great efforts from the scientific community, we believe that Au-based antibacterial nanostructures will show their potential as antibacterial agents in the coming days.

## Figures and Tables

**Table 1:** Summary of gold-based nanostructures and their antibacterial mechanisms.

No	Nanostructures	Antibacterial Mechanism	Bacteria	Reference
1	Cefotaxime (CTX)-loaded AuNP(C-AuNP)	The C-AuNPs prevented the peptidoglycan cell wall synthesis by inhibiting transpeptidation in the bacterial cell wall.	*S. aureus*, *E. coli*, *Klebsiella oxytoca*, *P. aeruginosa*	[51]
2	Ciprofloxacin- AuNCs(CIP:AuNP)	ROS generation and damage cell membrane	*S. aureus* and *E. coli*	[57]
3	Epigallocatechin gallate (EGCG) to hydrogels modified with AuNPs(E-Au@H)	PTT	*S. aureus*, *E. coli*, and *S. aureus biofilms*	[64]
4	Gold-decorated magnetic nanoparticles of Fe0 conjugated with sulfamethoxazole (MNPs@AuNPs/SMX)	Cell wall damage and ROS generation	*S. aureus* and *E. coli*,	[69]
5	Thioproline (T) and Boc-protected thioproline (B) in combination with AuNPs(TB-AuNPs)	Cell wall damage	*E. coli*, *MDR E. coli* and *Klebsiella pneumonia*	[75]
6	GSH-AuNCs	ROS generation	*Acetobacter aceti*	[84]
7	Phosphine-capped AuNCs	DNA damage and ROS generation	*MDR P. aeruginosa*	[85]
8	NHC-protected AuNCs	ROS generation	*S. aureus*, *E. coli*, and *C. albicans*	[86]
9	MHA-AuNCs	ROS generation, membrane damage, DNA damage	*S. aureus*, *E. coli*, *S. epidermidis*, *B. subtilis*, *C. difficile*, *F. nucleatum* and *P. aeruginosa*	[87,88,91,96,110]
10	DTAC-AuNCs/ICG	ROS generation, PTT	*MRSA*	[92]
11	Lys-AuNCs-Cur	ROS generation	*S. aureus*, *E. coli*, and *MRSA*	[93]
12	AuNC-ZwBuEt	ROS generation	*P. aeruginosa* and *S. aureus*	[91]
13	MUTAB- AuNCs	ROS generation	*E. faecalis*, *VRE*, *S. pneumoniae* and *P. aeruginosa*	[94]
14.	QA-AuNCs	Membrane damage, ROS generation, and disturbance	*MRSA*	[111]
15	Polyurethane consists of gold nanorods and polyethylene glycol (PU-Au-PEG)	PTT	*P. aeruginosa*, *S. aureus*, *MRSA*	[100]
16	AuNRs636, AuNRs772, AuNPs, and AgNPs	PDT (ROS generation) and PTT	*E. coli*, *S. aureus*, *Salmonella enterica*, and *MRSA*	[101]
17	MoS2@AuNRs	PDT (ROS generation) and PTT	*E. coli*	[103]
18	AuNRs-(200) and AuNBPs-(111)	PTT	*E. coli*	[105]
19	D/L-Glu-Au NBPs	Chemotherapy, physiotherapy and PTT	*S. epidermidis*	[106]
20	Gold nanostar (AuNSs)	Cell membrane damage and PTT	*S. aureus*	[107]
21	Vancomycin-modified gold nanostars (AuNSs@Van)	Cell wall damage and PTT	*methicillin-resistant Staphylococcus aureus*	[109]

## Data Availability

No new data were created or analyzed in this study. Data sharing is not applicable to this article.
